# Treatment algorithm in 960 pediatric burn cases: A review of etiology and epidemiology

**DOI:** 10.12669/pjms.345.15101

**Published:** 2018

**Authors:** Veli Avci, Omer Faruk Kocak

**Affiliations:** 1 Veli Avci, MD. Department of Pediatric Surgery, Yuzuncu Yil University, Medical Faculty, Van, Turkey; 2 Omer Faruk Kocak, MD. Department of Plastic, Reconstructive and Aesthetic Surgery, Yuzuncu Yil University, Medical Faculty, Van, Turkey

**Keywords:** Burn, Treatment, Algorithm, Etiology, Epidemiology

## Abstract

**Objective:**

Burn injuries are one of the most significant threats to life in both undeveloped and developing countries. In this study, we evaluate the demographic characteristics and treatment methods in pediatric burn cases admitted to our clinic.

**Methods:**

A total of 960 patients aged 0–16 years old who were referred to our center with burn injuries between 2015 and 2016 were analyzed in terms of sex, age, etiology, epidemiology, burn percentage, the degree of burn, hospitalization duration, morbidity-mortality, and treatment methods.

**Results:**

In the present study, 512 male and 448 female patients were included. Burns were seen mostly among the patients aged 2–4, and the majority of them were extremity burns. The mean hospitalization duration was 10±6.7 days, and the most common source of burn injury was from hot liquids.

**Conclusion:**

Burn injuries are a pediatric emergency that needs to be emphasized to reduce occurrences due to the long hospitalization period, the unfavorable mortality and morbidity rates, and increased treatment costs. It is possible to obtain more positive results by way of a standard and easily applicable treatment algorithm in cases of burn injury.

## INTRODUCTION

Burn injuries are more commonly seen in undeveloped societies where there is a low socio-economic status, and mostly in rural areas.^1^,^2^ In the United States, burns are one of the most common causes of mortality and morbidity among children^3^ Children are at greater risk since their defense mechanisms and reflexes are not fully developed.^4^

According to estimates of the World Health Organization (WHO), the number of children who died from burns was approximately 96,000 in 2004.^5^ The applied treatment method is an important issue that needs to be emphasized for such a trauma with high mortality and morbidity. Issues, such as the length of hospitalization, the agents used in treatment, the means of dressing and how often dressings are changed, and what should be done to prevent morbidity, differ from center to center. In this study, our aim was to evaluate the demographic characteristics and new treatment algorithms in pediatric burn cases.

## METHODS

**Fig.1:**
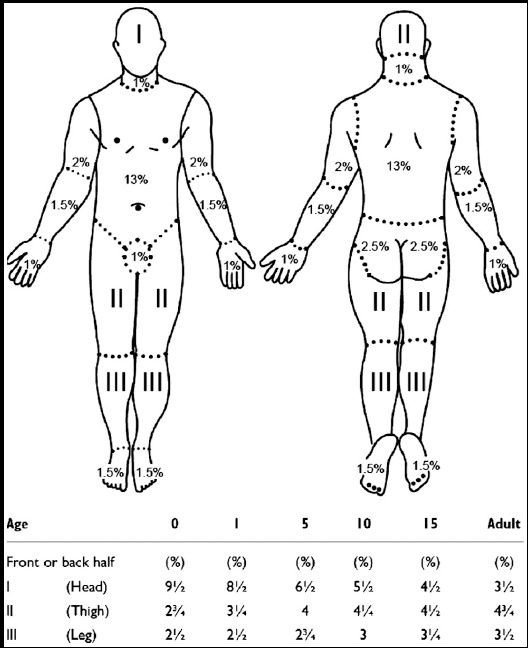
Lund-Browder Diagram

This study was carried out in accordance with the principles of the Helsinki Declaration. Local ethics committee approval number is 81966737-622.01. In the present study, the etiologic and epidemiological data of 960 patients aged 0–16 years that presented at our clinic due to burn injuries between 2015 and 2016 were retrospectively reviewed and recorded. A Lund-Browder classification was used to calculate the affected body surface area of the patients (Fig.1). Patients were divided into five groups according to total body surface area (TBSA) and four groups according to burn depth. Details of each patient in terms of sex, age, percentage and degree of burn, duration of hospitalization, morbidity-mortality and treatment methods were recorded. A new, easy, applicable treatment algorithm was developed for burn injuries. Electric burns were not included in the present study since no such cases presented at our clinic.

### Statistical Analyses

Continuous statistical variables were expressed as mean, standard deviation, minimum and maximum values, whereas categorical variables were expressed with numbers and percentages. A Chi-square test was used to determine the relationship between categorical variables, and a p-value of <0.05 was considered statistically significant. The data obtained from the statistical analyses were recorded and analyzed using SPSS version 10.0 software (IBM Corporation, Armonk, NY, USA).

## RESULTS

Of the 960 patients, 53% (n=512) were male and 47% (n=448) were female, and 78% (n=745) were included in the 2–4 year-old patient group. The mean age was 2.4 ± 1.5 (Table-I). No significant difference was observed between age groups and sex (p = 0.68). Of all patients, 85% (n=813) were living in rural areas and 15% (n=147) were living in urban areas.

**Table-I:** Patient distribution according to years.

Years	Cases
<1	6
Year 1-2	28
Year 2-3	381
Year 3-4	364
Year 4-5	98
Year 5-6	34
>6	49

For the degree of burn, 62% (n=596) of patients were in the severe burn group, whereas 38% (364) of patients were in the mild-moderate burn group (Table-II). According to Lund-Browder classification, the larger range of TBSA was 10-20% (n = 334, 35%) (Table-II). Increased burn depth and TBSA were associated with increased hospitalization and morbidity (p <0.05). According to the garnered data, burns were most commonly seen in the extremities (53%, n=504). Isolated genital area burns were very rare (0.002%, n=2), and 58 (0.06%) genital area burns were classed as combined burns. Head-neck-face burns accounted for 8% (n=76) (Table-III). Of all cases, 85% (n = 816) were in the group which included burns from hot water or tea, and 11% (n = 102) of cases were in the group that included burns from dairy products (Table-IV). No significant relationship was observed between hot water burns and gender (p>0.05), while a significant relationship was found between floor furnaces and dairy product burns and females (p<0.05).

**Table-II:** Patient distribution according to burn depth-TBSA

Burn Depth	Cases
1°	10
Superfical Burn2°	354
Deep Burn 2°	582
3°	14
TBSA	
1-10%	242
10-20%	334
20-40%	266
40-60%	95
>60%	23

**Table-III:** Patient distribution according to burn locations.

Burn Locations	Cases
Genital	58
Head-neck-face	76
Trunk	370
Ekstremities	580
Totally	960

**Table-IV:** Patient distribution according to burning type.

Burning Type	Cases
Hot water-Tea	816
Dairy products	102
Floor furnaces	36
Chemical Agents	6

The minimum hospitalization duration was five days, and the maximum was 34 days, with mean hospitalization duration of 10±6.7 days. A significant relationship was identified between the surface area and degree of the burn, the number of surgical interventions and the development of infections, and the number of days of hospitalization (p <0.05).

**Fig.2:**
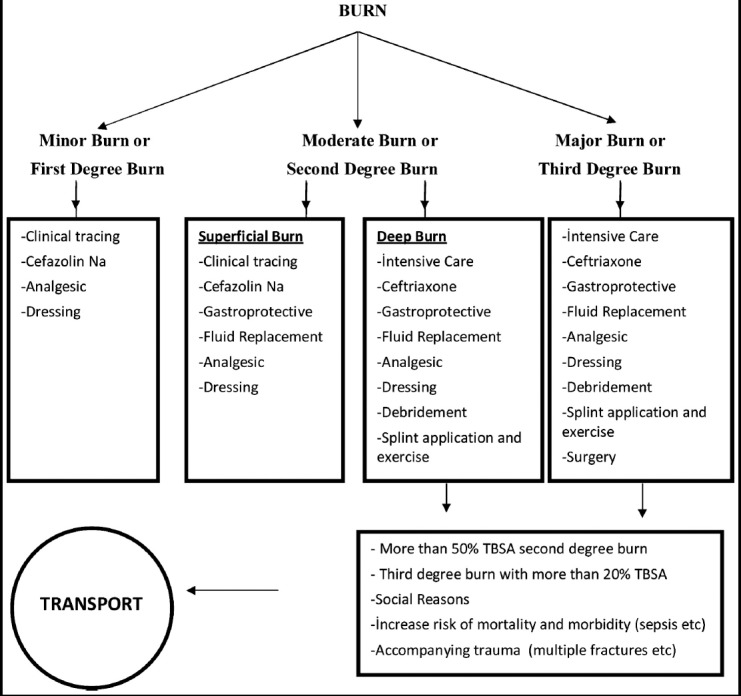
Treatment algorithm according to burn degree and the percentage. ***Classification of Burn Percentage:***. ***Minor Burns According to TBSA***. *First degree child burns *Second degree child burns less than 10% TBSA ***Moderate Burns According to TBSA***. *Second degree child burns involving 10 to 30% TBSA *Third degree child burns less than 10% TBSA ***Major Burns According to TBSA***. *In children, second degree burns greater than 30% TBSA *Third degree child burns greater than 10% * Burns of the eye, ear, face, hand, foot, and genitalia.

Secondary surgical interventions were applied to 14% (n = 134) patients. 64 contracture releasing (multiple Z-plasties), 32 scar revisions, 10 tenolysis-neurolysis, 25 flap-grafts applications and three nerve repair with nerve grafts were done. In open wounds, grafting was performed as the first choice but in the case of bone and tendon exposures, flap alternatives were preferred. One patient who underwent two sessions of grafting had a maximum length of stay of 34 days. A significant relationship was found between the type of burn and hospitalization duration, and a significant relationship was identified between burns due to hot water (p=0.02), dairy products (p=0.01) and floor furnaces (p=0.01), and the number of days of hospitalization. An algorithm was created in accordance with the treatment methods applied (Fig.2).

## DISCUSSION

In developing countries, burn injuries are one of the major public health problems, with incidences of one percent reported in the global population, and 0.79 percent in children.^1^ Toddler and preschool children are more sensitive concerning burns. According to WHO, burns rank 11th among the causes of child death, and are the fifth most common cause of non-fatal child injuries.^6^

Burn injuries may vary depending on the socio-cultural and socio-economic status of the country in question although burn injuries are more common in male children under the age of four in all communities.^1^,^2^,^4^,^6^ Similarly, in our study, burn injuries were found to be common particularly in male children aged 2–4 years although no statistically significant difference was found in terms of age and sex (p> 0.05).

Incidences of burns were found to be significantly higher in regions with low income, and in these regions, a significant relationship was observed between low education levels, large family and burns.^2^,^3^ The reason for a large number of burn cases presenting to our clinic over a two-year period is that our clinic is located in a rural area where people live in stove-heated houses.

The severity of the burn depends on the degree and percentage of the burned area.^7^ Special grades are used in the calculation of burn percentage. Adult burns can be approximately predicted in the commonly used nines rule. For children, however, the Lund-Browder surface calculation diagram is used for a more accurate calculation method considering the age range.^8^ In two separate studies; burns less than 10% of TBSA were reported in 64% of cases and mean TBSA was reported about 12%.^6^,^7^ In our study, burns less than 10% TBSA were 25%, and this ratio was lower than the literature. But the average TBSA was 17.5% and this ratio was higher than the literature. This shows that TBSA is higher in our patients despite the literature; which forced us to find an effective treatment algorithm.

The cause of the burn should be known in the determination of the treatment modality. Burns result from many factors, such as electrical current, thermal inhalation and radiation.^7^ In earlier studies on burns etiology carried out by many researchers, hot liquid burns have been found to be most common, whereas burns from direct contact with flames take second place.^1^,^3^,^7^,^9^-^11^ In our study, hot water and tea burns took first place (85%), with dairy product burns taking second place (11%), in contrast to many earlier studies in which direct flame burns take second place. This finding may be attributed to that the majority of our burn cases (85%) were from families living in rural areas where animal husbandry is common, and where the relationship between dairy products and floor furnace burns and females was found to be statistically significant (p <0.05).

As the degree and percentage of the burn increases in burn cases, the mortality rate due to skin integrity and an impaired immune response also increases. The most common cause of mortality is the infection although systemic inflammatory response syndrome and sepsis are other causes.^12^,^13^ In studies carried out by Elsous et al. and Li et al. burns were responsible for mortality in four and six patients, respectively.^6^,^7^ In our case series, no mortality was observed in any of our patients, despite the fact that 62% of our cases were in the severe burn group. The reason for this could be the early treatment and easily accessible location of our hospital. Antibiotics were started in all cases because the burn cases admitted to our center were dirty. Broad-spectrum antibiotics were administered for larger and dirtier wounds, and in this way, morbidity and mortality rates were decreased, supported by the early administration of antimicrobial agents.

The following measures are taken as a standard for patients admitted to burn centers: fluid resuscitation, immediate removal of clothing from the injured area, cleaning the wound, debridement and dressing application, and early oral feeding.^6^,^8^ Dressing frequency, antibiotic use, surgical treatment decision, exercise and discharge date are different for each center. There is a lack of consensus on the treatment of bullae. According to some researchers, the contents of the bullae should be aspirated and used as a dressing, while in other studies, metabolites in the bulla have been suggested to reduce wound healing and reported to be cleaned.^14^ In some studies, small vesicles have been left in place, while large vesicles are evacuated.^6^ In our study, we preferred not to touch any large or small vesicles except for infected or dirty bullae. The epidermis layer on the vesicles has been observed to act as a natural protective dressing, and we believe that low rates of infection may be related to this.

Infections observed at the burn site are the most important causes of mortality and morbidity, and so creams and ointments that contain topical antimicrobial agents have been used in the treatment of burn injuries for a long time.^10^,^14^,^15^ The most commonly used antiseptic ointment for burn treatment is silver sulfadiazine although this creates a pseudo-epithelium on the wound.^12^ In our clinic, silver sulfadiazine and nitrofurazone ointment are combined in the second dressing, with only silver sulfadiazine used in the first dressing, given that it contains a local anesthetic agent (lidocaine). This scab created by silver sulfadiazine is debrided. Closed dressings are applied with nitrofurazone ointment every day with the third dressing after debridement. In this way, the pain caused by both infections of the wound and frequent dressing, as well as pain-related psychological trauma, are prevented.

The location of the burn is also important when selecting a treatment. Closed dressings are not advised for facial burns, unlike all other body regions, although ointment is applied twice a day to prevent the wound from becoming dry.^14^ For this treatment, we applied the cream that contains neomycin sulfate at least twice a day for 76 patients.

In many burn centers, the number of patients who undergo a surgical procedure is high.^10^ In a study that included 400 cases, 41% of the patients were reported as having undergone surgery.^1^ In the present study, however, only 14 percent (n=134) of patients who required surgical interventions, despite our treatment algorithm, splint and exercise applications, underwent surgical procedures, which included scar revision, contracture opening, reconstruction with graft or flap.

### Limitations of the study

Electric burn cases have high morbidity and mortality rates and may involve multiple systems.^16^ Electric burns were not included in our study; as such cases were referred to other centers due to the accompanying traumas, which could be considered a limitation of the present study.

## CONCLUSION

Burn injuries may lead to serious physical and psychological sequellae in acute and chronic periods. Epidemiologic studies should be considered important regarding their findings that show the risk factors associated with burns. Although progress has been made in the treatment of burn injuries thanks to the recently developing technologies, we believe that the algorithm used in this study may guide many physicians in the treatment of patients with burn injuries, and may reduce the cost as well as the number of patients requiring surgical intervention.

### Authors’ Contribution

**VA** conceived, designed and did statistical analysis & editing of manuscript.

**OFK and VA** did data collection and manuscript writing.

**OFK** did review and final approval of manuscript.
